# Hippocampal Acetylcholine Depletion Has No Effect on Anxiety, Spatial Novelty Preference, or Differential Reward for Low Rates of Responding (DRL) Performance in Rats

**DOI:** 10.1037/bne0000072

**Published:** 2015-08

**Authors:** Stephen B. McHugh, Anna Francis, J. Devin McAuley, Amanda L. Stewart, Mark G. Baxter, David M. Bannerman

**Affiliations:** 1 Department of Experimental Psychology, University of Oxford; 2 Department of Psychology and Neuroscience Program, Michigan State University, and Department of Psychology, Bowling Green State University; 3 Department of Psychology, Bowling Green State University; 4 Department of Experimental Psychology, University of Oxford

**Keywords:** acetylcholine, hippocampus, medial septum, anxiety, spatial memory, behavioral inhibition

## Abstract

We investigated the role of the septo-hippocampal cholinergic projection in anxiety, spatial novelty preference, and differential reward for low rates of responding (DRL) performance. Cholinergic neurons of the rat medial septum (MS) and the vertical limb of the diagonal band of Broca (VDB) were lesioned using the selective immunotoxin, 192 IgG-saporin. Rats were then tested on several behavioral tests previously shown to be sensitive to either (a) hippocampal lesions or (b) nonselective MS/VDB lesions which target both cholinergic and γ-aminobutyric acid (GABA)-ergic projections, or both. Saporin lesions substantially reduced hippocampal cholinergic innervation, resulting in an absence of acetyl cholinesterase staining and markedly reduced choline acetyltransferase activity (mean reduction: 80 ± 5%; range: 50–97%). However, the saporin-lesioned rats did not differ from control rats in any of the behavioral tests. Thus we found no evidence from these lesion studies that the septo-hippocampal cholinergic projection plays an essential role in anxiety, spatial novelty preference, or DRL.

The hippocampus receives cholinergic and γ-aminobutyric acid (GABA)-ergic input from the medial septum (MS) and the vertical limb of the diagonal band of Broca (VDB; Köhler, Chan-Palay, & Wu, 1984; Lewis & Shute, 1967). Like hippocampal lesions, nonselective lesions of the MS/VDB (i.e., lesions destroying both cholinergic and GABA-ergic neurons) impair spatial memory, and reduce anxiety and impair behavioral inhibition (Bannerman et al., 2002; Bannerman, Matthews, Deacon, & Rawlins, 2004; Ellen, Wilson, & Powell, 1964; Herrmann, Poucet, & Ellen, 1985; Kelsey & Grossman, 1971, 1975; McHugh, Deacon, Rawlins, & Bannerman, 2004; Menard & Treit, 1996; Miyamoto, Kato, Narumi, & Nagaoka, 1987; Morris, Schenk, Tweedie, & Jarrard, 1990; Sinden, Rawlins, Gray, & Jarrard, 1986). Indeed, an influential theory states that the septum and hippocampus form an integrated functional unit: the septo-hippocampal system (Gray & McNaughton, 1983, 2000). This hypothesis suggests that MS/VDB input is crucial for normal hippocampal function.

The hippocampus plays an essential role in spatial learning and memory. Initially, it was assumed that the effects of nonselective MS/VDB lesions on spatial memory were due to disruption of the cholinergic, rather than the GABA-ergic, inputs to the hippocampus. However, selective cholinergic lesions of the MS/VDB have little or no effect on spatial memory performance (Baxter, Bucci, Gorman, Wiley, & Gallagher, 1995; Chappell, McMahan, Chiba, & Gallagher, 1998; Fletcher, Baxter, Guzowski, Shapiro, & Rapp, 2007; Kirby & Rawlins, 2003; McMahan, Sobel, & Baxter, 1997; Pang & Nocera, 1999; Torres et al., 1994; Vuckovich, Semel, & Baxter, 2004). This notwithstanding, cholinergic inputs to the hippocampus may be important for only a subset of the functions performed by the hippocampus. For example cholinergic MS/VDB lesions do impair latent inhibition (Baxter, Holland, & Gallagher, 1997), conditional learning (Janisiewicz, Jackson, Firoz, & Baxter, 2004), and context-place recognition memory (Easton, Fitchett, Eacott, & Baxter, 2011), so it is not that these lesions are completely without effect. Moreover, the hippocampus plays a role not only in spatial information processing, but also in nonspatial aspects of memory, and in anxiety, and preferences for novel stimuli (Bannerman, Rawlins et al., 2004). The effect of selective cholinergic MS/VDB lesions on these processes remains unknown. Here we made selective cholinergic lesions of the MS/VDB in rats using the immunotoxin 192 IgG-saporin and investigated their effects on two ethological anxiety tests, a test of spatial novelty preference, and DRL (differential reinforcement for low rates of responding).

The two anxiety tests used were the successive alleys (a modified version of the plus maze), and food neophobia (also known as novelty suppressed feeding). Each of these tests relies on an approach/avoidance conflict to generate anxiety and both tests are sensitive to nonselective lesions of the MS/VDB and neurotoxic lesions of the ventral hippocampus (Bannerman et al., 2002; Bannerman, Matthews et al., 2004; McHugh et al., 2004). These tests have been validated pharmacologically with benzodiazepines, which reduce anxiety in both tests (McHugh et al., 2004; Shephard & Estall, 1984).

The spatial novelty preference task capitalizes on rodents’ innate preference for novel over familiar spatial environments. In this task, the rat initially explores two arms of a three-arm maze (sample phase). After a delay period, during which the rat is removed from the maze, the rat is then allowed to explore all three arms (test phase). During the test phase, normal rats exhibit a spatial novelty preference by spending more time in the previously unexplored arm than the explored arms. This test of short-term spatial memory is sensitive to both hippocampal lesions (Sanderson et al., 2007) and temporary inactivation of the dorsal hippocampus with lidocaine (Howland, Harrison, Hannesson, & Phillips, 2008).

Hippocampal lesions also impair behavioral inhibition on tasks such as DRL, which tests rats’ ability to withhold responding (e.g., lever pressing) for a fixed interval in order to gain a reward. For example, during DRL-15, the first lever press is rewarded but the rat must then wait for at least 15 s between subsequent lever presses to gain further rewards. Premature responding is not rewarded and the 15-s timer is reset. Rats that learn to inhibit lever pressing are more efficient, gaining more rewards per lever press. Efficient DRL performance therefore requires both behavioral inhibition and accurate timing of the interval between presses. DRL is sensitive to both hippocampal lesions and nonselective MS/VDB lesions (Ellen et al., 1964; Kelsey & Grossman, 1971, 1975; Sinden et al., 1986). This might suggest that cholinergic inputs from the MS/VDB to the hippocampus are important for DRL performance. However, against this it was reported previously that the effects of scopolamine, a cholinergic antagonist, on DRL performance were similar in hippocampal-lesioned and nonlesioned control rats (Sinden et al., 1986), which could be taken as evidence to suggest that hippocampal ACh receptors may make little, if any, contribution on this task. Using the 192 IgG-saporin lesion approach, we will now specifically test the contribution of MS/VDB cholinergic projections to the hippocampus in DRL.

Given that it has been argued that cholinergic input is critical for normal hippocampal function (Drever, Riedel, & Platt, 2011; Hasselmo, 2006) and that the behavioral tasks we selected are all sensitive to nonselective lesions of the hippocampus and/or the MS/VDB, we predicted that cholinergic lesions of the MS/VDB would affect behavior in all of these tasks.

## Materials and Method

### Subjects

This study used 36 naïve male Lister-Hooded rats (Harlan Olac, Bicester, U.K.), weighing between 310 and 385 g at the start of the experiment. They were housed in a temperature- and humidity-controlled room under a 12-hr light–dark cycle (lights on 0700–1900). Testing took place during the light cycle. Rats were housed three per cage with ad libitum food and water unless otherwise specified. The experiments were conducted in accordance with the United Kingdom Animals Scientific Procedures Act (1986) under project license PPL 30/1989.

### Surgery

Before surgery, rats were randomly assigned to one of three groups. One group received selective ACh lesions of the MS/VDB using the immunotoxin 192 IgG-saporin (SAP; *n* = 14), a ribosome-inactivating factor coupled to a monoclonal antibody raised against the low affinity p75 nerve growth factor receptor, expressed by cholinergic neurons (Wiley, Oeltmann, & Lappi, 1991). Pilot experiments demonstrated substantial cholinergic cell loss (as indexed by acetylcholinesterase histochemistry) without GABAergic cell loss (as indexed by parvalbumin histochemistry) using a 0.1 μg/μl concentration of the toxin. A second group received infusions of vehicle solution (VEH; *n* = 12) at the same coordinates as the SAP group. The third group received no surgical treatment (UN-OP; *n* = 10).

Rats in the SAP and VEH groups were anesthetized with isoflurane (3–4% in 4L/min O_2_ for induction, 1.5–2% in 1L/min O_2_ for maintenance) and placed in a stereotaxic frame with the head level between bregma and lambda. An incision was made along the midline of the scalp, the skin was retracted and two holes were drilled either side of the sagittal suture at coordinates anterior-posterior (AP) = +0.45 mm and medial-lateral (ML) = ± 0.6 mm from bregma according to the atlas of (Paxinos & Watson, 1998). A 28-gauge Hamilton syringe with a modified 34-gauge cannula (Scientific Glass Engineering, Milton Keynes, U.K.) was used to infuse phosphate buffered solution (PBS, pH = 7.4) into the VEH group or 192 IgG-saporin (0.1 μg/μl concentration; Advanced Targeting Systems, San Diego, CA) into the SAP group at depths of DV = −7.8 mm and dorsal-ventral (DV) = −6.2 mm. The syringe was left in place for 30 s before infusing either PBS or 192 IgG-saporin at a rate of 0.05 μl/min. A total volume of 0.3 μl 192 IgG-saporin or PBS was infused at the DV = −7.8 mm position, and 0.2 μl into the DV = −6.2 mm position. The syringe remained in position for 9 min following the 0.3 μl infusions and 6 min following the 0.2 μl infusions to limit diffusion along the needle track. Analgesia (Meloxicam, ∼2 mg/kg; s.c.) was given before and after surgery. On completion of surgery all rats were sutured and given at least 14 days postoperative recovery before testing began.

### General Behavioral Procedures

Behaviors were assessed with the experimenter blind to the allocation of rats to each group. The order of testing is presented chronologically. For the anxiety and spatial novelty tests, one or two nonexperimental rats were placed on or into the apparatus shortly before testing to give it a consistent rat-like odor.

### Experiment 1: Spontaneous Locomotor Activity

Because hyperactivity can affect performance on anxiety, spatial novelty preference, and DRL, we first assessed spontaneous locomotor activity in MS/VDB lesioned rats and controls. Locomotor activity was examined in a set of hanging wire cages (39 cm long × 25 cm wide × 23 cm high; Arrowmight, Hereford, U.K.) containing horizontal photocell beams located 13 cm apart along the long axis of the cage. The total number of beam breaks and crossovers during the 120-min session were recorded on computer (Acorn Archimedes RISC PC 600 running Arachnid Activity Monitor, Cambridge Cognition, Cambridge, U.K.). A crossover was counted when the front and back beams were broken consecutively, indicating the animal had crossed the length of the cage. Rats were placed individually into the cages and all testing was performed in the dark.

### Experiment 2: Successive Alleys Test

The successive alleys test is based on the elevated plus maze (Pellow, Chopin, File, & Briley, 1985). The alleys are of increasingly anxiogenic character, which provides a graded measure of anxiety and avoids the problems associated with interpretation of time spent in the central square of the plus maze. The apparatus consisted of four successive wooden alleys (each 45-cm long). Section 1 was painted black, was 9.5-cm wide, and had three 28.5-cm-high walls. Section 2 was painted gray, was also 9.5-cm wide, and had two 2.5-cm-high walls. A step down of 1.5 cm led to Section 3, which was painted white, was 6.5-cm wide, and had two 0.5-cm-high walls. Section 4 was also painted white, was 3.5-cm wide, and had three 0.25-cm-high walls. The apparatus was positioned on a Table 0.7 m above the floor such that Section 1 was actually on the table, but Sections 2–4 extended out above the floor. Behavior was analyzed online and offline by means of a camera mounted above the maze. Rats were transported individually and allowed to habituate in a separate room for 10 min prior to testing. During testing rats were placed in Section 1 facing the back wall. The test lasted 300 s and latencies to cross into each section and total time spent in each section were recorded. If a rat fell from the maze it was immediately replaced at the junction between the section from which it fell and the preceding section.

### Experiment 3: Food Neophobia

Food neophobia, an established test of anxiety also known as hyponeophagia and novelty-suppressed feeding (Shephard & Estall, 1984), was assessed by measuring the latency to begin eating in three potentially anxiogenic situations (Bannerman, Matthews et al., 2004; McHugh et al., 2004). Anxiety was manipulated by providing novel foodstuffs and/or by testing the rats in unfamiliar rooms on novel apparatus. Rats were food deprived overnight prior to testing. On the day of testing, rats were transported individually to habituate in a separate room for 10 min before being taken to the testing room. Then each rat was brought into the testing room and placed in the apparatus, and the latency to begin eating was recorded (maximum of 300 s). The three tests measured latency to eat (a) Noyes 45-mg reward pellets (Formula A/I; P.J. Noyes, Lancaster, NH) on an elevated T maze, (b) Noyes pellets in a white plastic bucket, and (c) pieces of sweet corn (Green Giant Original Niblets) on a feeding table, and have been previously described in detail elsewhere (Bannerman et al., 2002; McHugh et al., 2004). Each test was carried out in a different room that was novel to the rats, and both the Noyes pellets and the sweet corn were novel to the rats at the start of these experiments.

### Experiment 4: Spatial Novelty Preference

Spatial novelty preference is a spontaneous, unrewarded exploration task that exploits rodents’ natural tendency to prefer novel over familiar spatial environments (Sanderson et al., 2007). Rats were transported individually and left to habituate in the test room for 1 min. After this time each rat was placed on an elevated black Y maze (73-cm high; each arm 70-cm long, 10-cm wide, with 3 walls 2.5-cm high) facing away from the center on a designated start arm that was the same for each rat. One arm, the “familiar” arm, was accessible, while the “novel” arm was blocked by an L-shaped wooden block (a 15.5-cm-long, 9-cm-high and 8-cm-wide piece of wood nailed to a 4.5-cm-long, 20-cm-high and 8-cm-wide piece of wood). Attached to the taller block was an aluminum sheet (31-cm wide, 40-cm high), which prevented the rat seeing into or gaining access to the novel arm. Allocation of arms as “familiar” or “novel” was counterbalanced across groups. When the rat left the start arm (defined as all four paws being outside the arm), the rat was permitted to explore for 5 min and the number of entries into the start arm and the familiar arm and the total time spent in both arms were recorded. This is known as the *sample phase*. The rat was then returned to its cage for 1 min. The block was taken away, fecal boli and/or urine were removed and the maze cleaned. The rat was then once again placed on the start arm. The *test phase* started when the rat left the start arm. The rat was now permitted to explore freely all three arms for 2 min, during which the number of entries and total time spent in each arm were again recorded. Three rats failed to leave the start arm at all and so were excluded from subsequent analysis.

### Experiment 5: Differential Reinforcement for Low Rates of Responding (DRL-15)

DRL was performed in one of eight identical operant chambers (30 × 24 × 21 cm; Med Associates, St. Albans, VT) housed in sound attenuating cabinets. Each chamber contained a central food magazine and a retractable lever. The magazine detected head entries via an infrared beam and could be illuminated by an internal light. Each chamber was illuminated by a 2.8 W house light and pellet delivery, lever presses and food magazine entries were controlled by a PC running Med-PC software (WMPC version IV, Med Associates).

Rats were food restricted until they reached 85% of their free-feeding weights and were then strictly maintained at this 85% level throughout the DRL experiment. Rats received one training session per day. They were first habituated to the operant chambers for 10 min, with 20 food pellets (Formula A/I; P.J. Noyes) in the magazine tray. They then received two 30-min sessions with a variable interval 16-s schedule. There followed eight 15-min sessions of fixed-ratio (FR) training, during which the rats learned to lever-press for reward (2 × FR1, 2 × FR2, 2 × FR4, 2 × FR8). During the FR1 sessions, breakfast cereal pieces (Shreddies™, Nestle, Surrey, U.K.) were taped to the response lever to encourage pressing.

DRL training began on Day 12 and rats received 16 DRL sessions. Each session began with the onset of the house light and the first lever press was rewarded with a single food pellet. Thereafter, lever presses made 15 s or more after the previous response were rewarded with a single food pellet; responses made before the 15 s had elapsed were not rewarded and the timer was reset. The session ended after 30 mins with the offset of the house light. The number of rewards earned and the number of lever presses made were counted and the efficiency of the rats was calculated as the ratio of these two measures (efficiency = rewards/lever presses). We also measured the temporal distribution of responses (interresponse time, IRT) in 3-s time bins from the time of the previous response (12 time bins from 0–36 s, plus one time bin for responses after 36 s). For clarity, we report IRT data from a single day (DRL Session 11) when rats in all groups were at near-maximum efficiency.

After the 16 drug-free sessions, rats received four additional DRL-15 sessions, in which the effects of the benzodiazepine chlordiazepoxide (CDP, 4 mg/kg, i.p.) were assessed using a counterbalanced within-subjects design. CDP has previously been shown to impair DRL efficiency (Panickar & McNaughton, 1992; Sanger, 1980), and here we investigated whether this effect was potentiated following SAP-lesions. Each rat received two drug injection sessions (A) and two vehicle injection sessions (B) in an ABBA design (50% of the rats in each group) or BAAB design (the other 50%). Data from drug (A) versus vehicle (B) sessions were combined and analyzed with ANOVA (model: lesion group_3_ × (drug condition_2_ x S_36_)).

### Histology

#### Acetylcholinesterase histochemistry

At the end of the experiments, nine rats (SAP: *n* = 3; VEH: *n* = 3; UN-OP: *n* = 3) were injected with Euthatal (200 mg/ml sodium pentobarbitone; 200 mg/kg i.p.) and perfused transcardially with 0.9% heparinized saline, followed by 350 ml 4% paraformaldehyde (PFA). The brains were removed and postfixed in 4% PFA for 24 hours, transferred to 0.1M PBS/0.1% sodium azide until 2–3 days before sectioning, then cryoprotected in 30% sucrose/PBS and stored at 4 °C. On the day of sectioning, brains were frozen and 50 μm coronal sections were cut using a base-sledge microtome. Sections were processed for acetycholinesterase (AChE) histochemistry using the method of Karnovsky and Roots (1964). Briefly, following overnight incubation (100 ml of stock solution consisting of 250 ml deionized water, 1.7 g sodium acetate, 0.25 g copper sulfate, and 0.3 g glycine added to 116 mg acetylthiocholine and 3 mg ethopropazine), sections were developed in sodium sulfide (200 ml 0.1M acetic acid, 2 mg sodium sulfide). Sections were mounted on glycerine/albumin coated slides and left to air dry. Sections were intensified by silver nitrate, dehydrated in increasing concentrations of absolute alcohol (70%, 80% and 90%), and then cover-slipped.

#### Choline acetyltransferase (ChAT) assay

The remaining 27 rats (SAP: *n* = 11; VEH: *n* = 7; UN-OP: *n* = 9) were sacrificed using CO_2_, decapitated, and their hippocampi rapidly dissected and frozen. This tissue was then processed for determination of choline acetyltransferase (ChAT) activity in order to provide a quantitative assessment of the degree to which cholinergic denervation had been achieved.

To determine ChAT activity, tissue samples were homogenized with Tris/Triton solution (1:20 dilution, containing 0.05M Trizma HCl, 0.05M Trizma Base, and 0.02% Triton-X-100, pH 7.6) and 1.0 mm glass disruption beads and then centrifuged for 10 min. The resulting supernatant was pipetted into a separate microcentrifuge tube and subjected to a second centrifuge for 10 min in order to resediment any remaining macromolecules. The formation of [14C]Acetylcholine from [acetyl-1-14C]-acetyl-coenzyme A was used to measure the activity of ChAT, which synthesizes acetylcholine. A reaction mixture (20 μl) containing 0.134 mM 14C-Acetyl-CoA (60 mCi/mmol; MP Biomedicals, Irvine, CA) and 2.06 mM unlabeled compound was added to triplicate assay tubes already containing supernatant (20 μl) and a buffer (70 μl) consisting of 300 mM NaCl, 8 mM choline bromide, 20 mM EDTA, 0.1 mM eserine sulfate, 0.5% Triton X-100, and 50 mM dibasic sodium phosphate (pH 7.4). The tubes were vortexed and incubated at 37 °C for 30 min. The reaction was terminated by adding 5 mL 0.1 M cold phosphate buffer (pH 7.4). To measure ChAT activity, labeled acetylcholine was extracted by transferring the contents of the tube to a scintillation vial and adding 10 mL of toluene (containing 15*g*/L PPO and 1*g*/L POPOP) and 2 mL acetonitrile (containing 5*g*/L Na-tetraphenylboron). The vials were allowed to settle for 12 hours before the organic phase (top layer) was counted with a scintillation counter. The protein content of each sample was calculated according to the method described in Bradford (1976) using a Bio-Rad (Bio-Rad Laboratories, Hercules, CA) protein assay. Supernatant was diluted to 1:200 with Tris/Triton solution. Ten μl diluted supernatant was pipetted into triplicate wells of flat-bottom assay plates. Absorbance was measured at 595 nm. Protein content of the homogenates was calculated from standard curves that were prepared using serial dilutions of bovine serum albumin. ChAT levels, averaged across the left and right hippocampi, are reported as nmol/mg of protein/hr.

### Statistical Procedures

Data were analyzed using *t* tests or analysis of variance (ANOVA) in SPSS (version 15, SPSS Inc, Chicago, IL) or SigmaStat (SPSS Inc, Chicago, IL). Data that did not meet parametric assumptions were analyzed using nonparametric tests. Post hoc pairwise comparisons were performed with appropriate corrections to keep the familywise error-rate at α = .05.

## Results

### Histology

**Figure 1 fig1:**
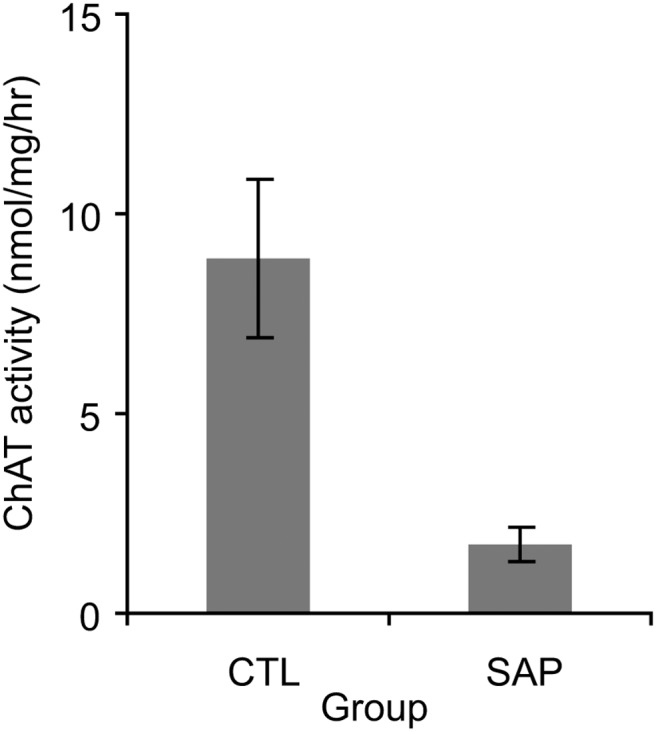
Mean (±standard error of the mean) hippocampal choline acetyltransferase (ChAT) activity in control (CTL) rats (combined unoperated and vehicle-infused) and rats with saporin (SAP) lesions.

Hippocampal AChE staining was virtually absent in the SAP group compared to the VEH and UN-OP groups, and the spatial distribution of AChE depletion was consistent with the description in Baxter et al. (1995; data not shown). Furthermore, hippocampal ChAT activity was significantly lower in the SAP group than the controls (Kruskal-Wallis ANOVA on ranks: H(2) = 16.1, *p* < .001; Dunn’s post hoc pairwise comparisons: SAP vs. UN-OP, *p* < .05; SAP vs. VEH, *p* < .05; VEH vs. UN-OP, *p* > .9). ChAT activity was reliably obtained from 26 rats (SAP: *n* = 10; VEH: *n* = 7; UN-OP: *n* = 9). Across the VEH and UN-OP rats, mean ChAT activity levels were 8.8 ± 2.0 nmol/mg of protein/hr. ChAT activity in SAP lesioned rats ranged from 0.2–4.4 nmol/mg of protein/hr (mean: 1.8 ± 0.4 nmol/mg of protein/hr, i.e., 2.5–49.3% of control levels; see Figure 1). In short, the SAP group had hippocampal cholinergic depletions ranging from 50–97.5% of controls (mean: 80.2 ± 4.9% depletion).[Fig-anchor fig1]

### Locomotor Activity

Locomotor activity decreased in all rats over the 2-hour session, but there were no group differences in the total number of cross-overs, *F*(2, 33) < 1; *p* = .4, or beam breaks, *F*(2, 33) < 1, *p* = .6. Analysis of beam break activity divided into six 20-min time bins (ANOVA: Lesion Group_3_ × [Time Bin_6_ × S_36_]) revealed a main effect of time bin, *F*(5, 165) = 123; *p* < .0001, but no Lesion Group × Time Bin interaction, *F*(10, 165) = 1.5, *p* = .1. Within the SAP group, ChAT levels did not correlate significantly with either beam-breaks (*r* = −0.3, *p* = .3) or crossovers (*r* = −0.1, *p* = .8). Thus, cholinergic lesions of the MS/VDB did not result in changes in locomotor activity.

### Successive Alleys

The SAP group did not show reduced anxiety compared to either the VEH or UN-OP groups on the successive alleys test. Latencies to cross into the more anxiogenic arms were similar for the three groups (all H(2) < 1.2, all *p* > .2; see Table 1). The amount of time spent in each section also did not differ among the three lesion groups (no Lesion Group × Section interaction), *F*(4, 66) < 1, *p* = .9 (see Table 2). Within the SAP group, ChAT levels did not significantly correlate with latency to leave the first alley (*r* = −0.5, *p* = .1) or time spent in the first alley (*r* = .1, *p* = .8). In short, there was no evidence that hippocampal cholinergic depletions affected anxiety in this test.[Table-anchor tbl1][Table-anchor tbl2]

### Food Neophobia

**Figure 2 fig2:**
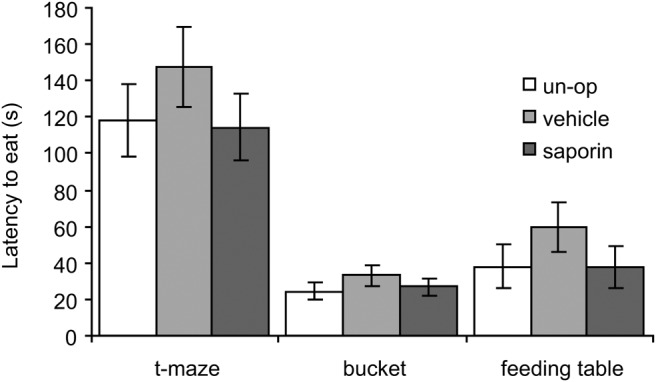
Mean (±standard error of the mean) latency to eat on the three food neophobia tests of anxiety for unoperated (un-op), vehicle infused and saporin-lesioned rats. There were no group differences in any of the tests.

There was no evidence that the SAP group were less anxious in any of the three food neophobia tests either (see Figure 2). Analysis (ANOVA: Lesion Group_3_ × [Test_3_ × S_36_]), showed a main effect of test, *F*(2, 66) = 53.4; *p* < .05, reflecting longer latencies to eat on the T maze test than in the bucket or feeding table tests (both *p* < .001). But there was no effect of lesion group, *F*(2, 33) = 1.4; *p* > .2, or Lesion Group × Test interaction, *F*(4, 66) < 1; *p* > .2. Within the SAP group, there was no significant correlation between ChAT levels and latency to eat (*r* = −0.1, *p* = .8). Thus, again, there was no effect of hippocampal cholinergic depletions in this anxiety test.[Fig-anchor fig2]

### Spatial Novelty Preference Test

**Figure 3 fig3:**
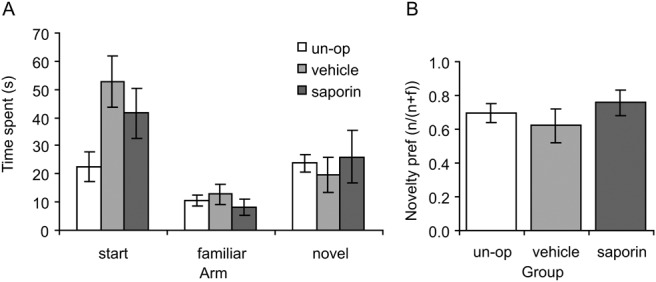
Spatial novelty preference. (A) Mean (± standard error of the mean) time spent in the three arms of a y-maze during the test phase of the task. There were no differences between the groups in terms of time spent in the novel or familiar arms. Note that the unoperated (un-op) group spent less time in the start arm than the other two groups, but this was because the un-op group spent more time in the “central area” (i.e., at the choice point). (B) Mean (± standard error of the mean) preference for the novel arm expressed as a ratio (novel/[novel + familiar]). There were no group differences on this measure.

In the spatial novelty preference test, rats spent significantly more time exploring the novel arm than the familiar arm during the test phase, and this novelty preference was equivalent in the three groups (see Figure 3). There was no evidence of impaired spatial novelty preference in the SAP group. (Note that the reduced time spent in the start arm by the UN-OP group versus VEH or SAP was because UN-OPs spent more time in the central area [i.e., the section of the Y maze where all three arms met].) Analysis (ANOVA: Lesion Group_3_ × [Arm_(novel vs. familiar)2_ × S_33_]) revealed a main effect of arm, *F*(1, 30) = 9.7; *p* < .005, with more time spent in the novel than the familiar arm, but no Lesion Group × Arm interaction, *F*(2, 30) < 1, *p* = .6. In addition, because time spent in the novel and familiar arms are not strictly independent of one another, we calculated a preference index based on time spent in the novel (*n*) arm divided by time spent in the novel and familiar (f) arms combined (*n*/(*n* + f)). One-way ANOVA on this preference index found no group differences, *F*(2, 30) < 1, *p* = .5, with both the SAP group and the controls exhibiting significantly stronger preference for the novel arm than would be expected by chance (*t* > 2.9, *p* < .01). Within the SAP group, there was no significant correlation between ChAT levels and the novelty preference index (*r* = .25, *p* = .6).[Fig-anchor fig3]

### Differential Reward for Low Rates of Responding (DRL-15)

**Figure 4 fig4:**
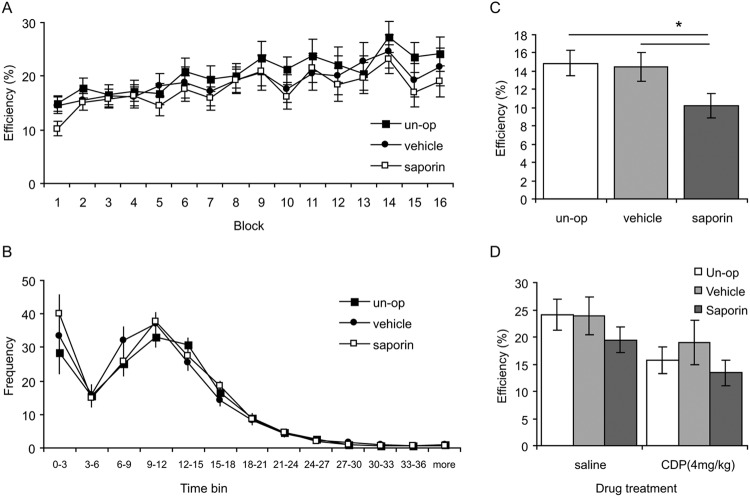
Differential reward for low rates of responding (DRL-15). (A) Mean (±standard error of the mean [*SEM*]) efficiency in DRL-15 training. Efficiency is computed as [number of rewards/number of lever presses]. Overall, all groups improved their efficiency at the same rate. (B) Interresponse time expressed as frequency of first lever press after reward delivery, in 3-s time bins. The shape of the response curves was equivalent in all three groups. (C) Mean (±*SEM*) efficiency during the very first block of DRL-15 training. Saporin lesioned rats were significantly less efficient than either the unoperated (un-op) or vehicle controls (* *p* < .05). (D) Mean (±*SEM*) efficiency following benzodiazepine (CDP) or saline injections. CDP impaired efficiency in all groups to an equivalent extent.

All groups improved their efficiency over the 16 blocks of DRL-15 training at a roughly equivalent rate (Figure 4A). Analysis (ANOVA: Lesion Group_3_ × [Block_16_ × S_36_]) revealed a main effect of block, *F*(15, 495) = 9.8, *p* < .001, but no main effect of lesion group, *F*(2, 33) < 1, *p* = .6, or interaction, *F*(30, 495) < 1, *p* = .98. Within the SAP-group, there was no correlation between ChAT levels and mean DRL efficiency (*r* = .06, *p* = .9). Moreover, examination of the temporal distribution of lever presses showed equivalent timing curves in all three groups (Figure 4B). Analysis (ANOVA: Lesion Group_3_ × [Time Bin_13_ × S_36_]) revealed a main effect of time bin, *F*(12, 396) = 92.8; *p* < .001, but no effect of lesion group, *F*(2, 33) < 1, *p* = .6, or Lesion × Block interaction, *F*(24, 396) < 1, *p* = .6. In short, cholinergic lesions did not affect DRL performance when analyzed across the 16 training blocks.[Fig-anchor fig4]

However, during the very first DRL session, rats in the SAP group were less efficient than both control groups (see Figure 4C). A one-way ANOVA restricted to just this first DRL session revealed a main effect of lesion group, *F*(2, 33) = 3.6, *p* = .04, reflecting lower efficiency in SAP group compared to both control groups (pairwise comparisons for SAP vs. UNOP: *p* = .02; SAP vs. VEH: *p* = .04). Within the SAP group, ChAT levels did not correlate with efficiency during the first training block (*r* = .3, *p* = .4), arguing that the extent of impairment was not dependent on the extent of cholinergic depletion, at least within our range of depletions (50–97.5%).

DRL efficiency is impaired by benzodiazepines in normal rats (Panickar & McNaughton, 1992; Sanger, 1980) and here we investigated whether this impairment was potentiated in the SAP-lesioned rats. However, benzodiazepines (CDP, 4 mg/kg, i.p.) impaired efficiency in the SAP and control groups to an equivalent extent (Figure 4D). Analysis of efficiency (ANOVA: Lesion Group_3_ × [Drug_2_ × S_36_]) revealed a main effect of drug, *F*(1, 33) = 22.4, *p* < .001, but no effect of lesion group, *F*(2, 33) = 1.0, *p* = .4, and no interaction, *F*(2, 33) < 1, *p* = .6. Within the SAP group, there was no correlation between ChAT levels and DRL efficiency, either under saline (*r* = −.05, *p* = .9) or CDP (*r* = −0.1, *p* = .8). In short, the ability of benzodiazepines to impair DRL efficiency was not affected by cholinergic lesions of the MS/VDB.

## Discussion

### Summary of Results

Despite substantial hippocampal cholinergic depletion (∼80%), the saporin-lesioned group did not differ from control rats in tests of locomotor activity, anxiety, spatial novelty preference, or DRL. These findings are important because neurotoxic hippocampal lesions or nonselective lesions of the MS/VDB (destroying both cholinergic and GABA-ergic neurons), or both, reduce anxiety, impair novelty preference, and impair DRL performance. We conclude, therefore, that on the basis of these lesion studies hippocampal cholinergic inputs do not play an essential role in these behaviors.

### Anxiety

The hippocampus plays an important role in anxiety (Bannerman, Rawlins et al., 2004; Bannerman et al., 2014; Gray & McNaughton, 2000). Hippocampal lesions reduce anxiety (Blanchard & Blanchard, 1972; Deacon, Bannerman, & Rawlins, 2002), and this effect has been localized to the ventral hippocampus (Bannerman et al., 2002, 2003; Kjelstrup et al., 2002; McHugh et al., 2004; McHugh, Fillenz, Lowry, Rawlins, & Bannerman, 2011; Trivedi & Coover, 2004). In addition, manipulations of hippocampal ACh levels and intrahippocampal administration of ACh receptor ligands or AChE inhibitors appear to modulate anxiety (Degroot & Treit, 2002; File, Gonzalez, & Andrews, 1998; File, Kenny, & Ouagazzal, 1998), suggesting that cholinergic input to the hippocampus is important for anxiety. However, the present data fail to find support for an essential role for hippocampal cholinergic inputs in anxiety: there was no evidence that selective cholinergic lesions of the MS/VDB reduced anxiety despite producing an 80% reduction in ChAT activity, and a loss of AChE staining. Importantly, the present study used identical testing protocols as both McHugh et al. (2004), who found that neurotoxic ventral hippocampal lesions reduced anxiety, and Bannerman et al. (2004), who found that electrolytic MS/VDB lesions reduced anxiety. This discrepancy suggests that either the GABA-ergic projection from MS/VDB to hippocampus is essential for anxiety or that there is some redundancy in the GABA-ergic and cholinergic projections such that effects are only present when both pathways are disrupted.

Our results are broadly consistent with those of Lamprea and colleagues, who found little effect of intraseptal 192 IgG-saporin lesions on classic measures of anxiety in either the elevated plus maze (Lamprea, Cardenas, Silveira, Morato, & Walsh, 2000), or the open field (Lamprea, Cardenas, Silveira, Walsh, & Morato, 2003). In contrast, they did demonstrate reduced anxiety on the elevated plus maze following lidocaine infusions into the medial septum which will result in a temporary inactivation of both cholinergic and GABA-ergic neurons, thus potentially consistent with results from nonselective MS/VDB lesions (Lamprea, Garcia, & Morato, 2010). Lamprea et al. (2010) did, however, find differences between their intraseptal 192 IgG-saporin lesioned animals and controls during a second exposure to the plus maze, although it is important to note that performance during a second exposure to the test is likely to reflect different psychological processes from those activated during a first exposure (see File, Gonzalez, & Gallant, 1998 for discussion). Lamprea et al. (2010) also reported differences in exploratory activity levels between groups using a minute-by-minute analysis of the first 5-min exposure to the elevated plus maze. They attributed these effects on the elevated plus maze to differences in the initiation of exploratory activity, and the acquisition and retention of spatial information. We found no evidence for similar effects in the present study, either during the test of spontaneous locomotor activity which examined exploratory activity in a novel cage environment (Experiment 1), or during the spatial novelty preference test which provides a stimulus-specific assay of spatial short-term memory (STM) for recently experienced locations (Experiment 4). One possible explanation for this apparent discrepancy between Lamprea et al. (2010) and the present experiments is the much higher dose of saporin used in the former study (237.5 ng in a 0.5 μl injection volume; see below for further discussion).

### Spatial Novelty Preference

Thus, in the present study we found that cholinergic lesions of the MS/VDB did not affect spatial novelty preference. In marked contrast, neurotoxic hippocampal lesions produce profound deficits in this task, with lesioned animals showing no preference for the novel over the familiar arm during the choice phase (Sanderson et al., 2007). In contrast, in the present study, rats with selective cholinergic lesions of the MS/VDB exhibited just as strong a preference for the novel arm as nonlesioned controls.

Spatial novelty preference likely reflects a form of spatial STM which may also underpin spatial working memory performance on “win-shift” maze tasks. In this respect, the failure to observe a deficit in the present study is consistent with a number of previous reports that cholinergic lesions of the MS/VDB lesions do not affect spatial working memory. For example, “win-shift” spatial working memory performance on both water and land-based radial mazes and during rewarded alternation on the T maze is unimpaired following selective cholinergic lesions of the MS/VDB (Chappell et al., 1998; Kirby & Rawlins, 2003; McMahan et al., 1997).

However, two studies have reported spatial working deficits following cholinergic lesions of the MS/VDB. For example, Chang and Gold (2004) found impairments in spontaneous alternation and Shen, Barnes, Wenk, and McNaughton (1996) found impairments in a working memory radial maze task. It is difficult to reconcile these findings with those of the present study, and with the other studies cited above but the concentration of 192 IgG-saporin could be important. In our pilot studies we found that injecting a 0.2 ug/μl concentration of the toxin caused damage not only to cholinergic cells but also to GABA-ergic cells in the MS/VDB. Notably, Shen et al. used a 0.2 ug/μl concentration and Chang & Gold used a 0.5 ug/μl concentration, although neither study reported whether GABA-ergic cells in the MS/VDB were affected. In the absence of such data, their results are difficult to interpret.

### DRL (Differential Reward for Low Rates of Responding)

Hippocampal lesions and nonselective lesions of the MS/VDB cause profound impairments in DRL performance (Ellen et al., 1964; Kelsey & Grossman, 1971, 1975; Sinden et al., 1986). As mentioned previously, cholinergic antagonists such as scopolamine also impair DRL performance (Kelsey & Grossman, 1975; Sinden et al., 1986). However, scopolamine administration had similar effects on hippocampal lesioned and nonlesioned control rats, impairing efficiency and flattening the temporal distribution of responses in both groups (Sinden et al., 1986). This could be taken to suggest that ACh receptors outside the hippocampus contribute to the scopolamine-induced deficit in DRL. The results of the present study are potentially consistent with this. Selective cholinergic lesions of the MS/VDB did not affect DRL performance, either in terms of efficiency or in the temporal distribution of responses. Also, efficiency was impaired by benzodiazepine administration in saporin-lesioned and control rats to an equivalent extent. This argues for a very limited role for hippocampal acetylcholine in DRL performance.

However, it is worth pointing out that we did observe one effect of saporin lesions on DRL. During the very first training session, rats with saporin lesions were less efficient than either of the control groups, although this deficit was not evident in any of the other training blocks. Transient deficits in spatial working memory following MS/VDB saporin lesions have been reported previously. For example, Vuckovich et al. (2004) found a deficit during the first two sessions of postoperative training on the radial maze, followed by recovery of performance to control levels. In the case of DRL performance in the present study, it is possible that the lower efficiency in the very first session may reflect a reduced ability to adapt to the change from the fixed ratio schedule of responding (FR8) that rats performed during the previous two sessions before DRL training began. Such transient deficits are consistent with the idea that cholinergic lesions may lead to reduced behavioral flexibility rather than cause specific memory impairments. However, the precise cause of this behavioral inflexibility, and the underlying psychological processes that are disrupted in these animals, remain to be established.

### So What Does the MS/VDB ACh Projection to the Hippocampus Do?

There is no clear consensus on the role of the MS/VDB cholinergic projection to the hippocampus. At a physiological level, selective cholinergic MS lesions reduce the power, but not the frequency, of the hippocampal theta rhythm (Lee, Chrobak, Sik, Wiley, & Buzsaki, 1994). Although it has been argued that theta rhythm is important for spatial learning and memory, much of this evidence is correlational rather than causal (Colgin, 2013). Moreover, GABA-ergic projections from the MS/VDB may also be important for hippocampal theta (Yoder & Pang, 2005). Indeed, a recent reevaluation of the role of MS/VDB projections to hippocampus has argued that neither the ACh nor the GABA-ergic projection alone is critical for hippocampal-dependent spatial learning and memory and that impairments arise only when both ACh and GABA-ergic cells are destroyed (Baxter & Bucci, 2013; Pang, Jiao, Sinha, Beck, & Servatius, 2011; Pang & Nocera, 1999). Comparison of nonselective and cholinergic MS/VDB lesion effects is potentially consistent with this hypothesis. However, it is also important to consider potential limitations of the immunotoxic lesion approach.

### Limitations of 192 IgG-Saporin Cholinergic Lesions

The depletions of ChAT activity in the current study were substantial (∼80% on average), but it remains possible that the spared fibers provide sufficient cholinergic input to the hippocampus to support behavioral performance. Indeed, it has been reported previously that despite a near total loss of ChAT positive neurons in the hippocampus following 192 IgG-saporin lesions, there was only a 40% decrease in ACh release in the hippocampus measured by dialysis. This suggests significant residual cholinergic function in the hippocampus of these animals (Chang & Gold, 2004). We cannot rule out the possibility therefore that a more complete depletion of ACh might have resulted in behavioral impairments. However, it is also worth noting that we found no evidence of any correlation between the extent of ACh depletion and behavioral performance in any of the present set of experiments. Thus, there was no suggestion that animals with greater than 80% depletion were more affected on the tasks. Along similar lines we also cannot completely exclude the possibility that the order in which the tasks were run, and thus prior experience on an earlier task, could have negated the effects of the saporin lesions on subsequent task performance.

Alternatively, some behavioral deficits may only emerge when cholinergic neurons beyond the MS/VDB are lesioned. For example, Wiley and colleagues found that working memory on the radial maze was only impaired with intracerebral ventricular 192 IgG-saporin lesions affecting 75% of total ACh neurons in the basal forebrain. Even when a significant portion of hippocampal-projecting ACh neurons were damaged, working memory was not impaired (Wrenn, Lappi, & Wiley, 1999).

The extent of depletion obtained in the present study may be near the limits of what is achievable with 192 IgG-saporin without causing unintended damage to GABA-ergic cells within the MS/VDB. It is also possible that adaptive, compensatory changes occur during the recovery period after the lesions which mask any potential behavioral phenotype. For example, in contrast to the present study, the importance of hippocampal ACh for anxiety is suggested by studies showing anxiogenic/anxiolytic effects of ACh ligands infused directly into the hippocampus (File, Gonzalez, & Andrews, 1998; File, Kenny, et al., 1998). These acute manipulations of cholinergic transmission may avoid any compensatory changes that might result from the chronic depletion of the neurotransmitter after a saporin lesion. For example, the effects of ACh on anxiety seem to be mediated via serotonin (File, Kenny, & Cheeta, 2000; Kenny, Cheeta, & File, 2000). It is possible that, following chronic ACh depletion, the serotonergic system adapts to compensate for the change in ACh input, although direct evidence here is lacking. Nevertheless, acute changes in ACh levels may thus modulate this serotoninergic response in a way that does not occur after permanent ACh depletion.

Similarly, infusing the ACh ligands directly into the hippocampus or MS/VDB leads to spatial memory deficits (Blokland, Honig, & Raaijmakers, 1992; Bunce, Sabolek, & Chrobak, 2004; Elvander et al., 2004). However, spatial memory impairments are present following both increases and decreases in hippocampal ACh (Bunce et al., 2004; Elvander et al., 2004), suggesting that ACh disruption may be the key factor, rather than ACh depletion per se. With permanent cholinergic depletion, compensatory changes may occur that allow for essentially normal hippocampal function. A future strategy would be to use optogenetic approaches to target hippocampal ACh input in a temporally specific manner (Witten et al., 2010).

## Conclusion

In this study, substantial cholinergic depletions of the hippocampus were without effect in ethological tests of anxiety, spatial novelty preference or DRL. One possible conclusion is that hippocampal acetylcholine is not essential for these functions. However, given previous results with acute pharmacological manipulations of acetylcholine neurotransmission, we would not exclude the possibility that adaptive, compensatory changes occurring during the recovery period after the lesions may mask any behavioral phenotypes.

## Figures and Tables

**Table 1 tbl1:** Effects of Cholinergic Depletion in the Hippocampus on Latency to Enter Each Alley of the Successive Alleys Test of Anxiety

Measure	Group			Stats
Latency to enter alley (s)	UN-OP	VEH	SAP	Kruskal-Wallis
2	34.5 (18–122)	29 (24–45)	32 (18–41)	H(2) = .20 *p* > .20
3	159 (89.5–300)	114 (65–300)	300 (70–300)	H(2) = 1.09 *p* > .20
4	300 (284.5–300)	300 (223–300)	300 (300–300)	H(2) = 1.23 *p* > .20
*Note*. UN-OP = unoperated; VEH = vehicle solution; SAP = saporin. The latency to enter each alley is presented as (median ± interquartile range) in seconds. The data were analyzed using three separate non-parametric Kruskal-Wallis one-way ANOVAs on ranks. There was no main effect of group on the latency to enter any alley.				

**Table 2 tbl2:** Effects of Cholinergic Depletion in the Hippocampus on the Time Spent on Each Alley of the Successive Alleys Test of Anxiety

Measure	Group		
Total time spent on each alley (s)	UN-OP	VEH	SAP
1	213.1 ± 13.8	206.4 ± 16.0	224.1 ± 16.1
2	66.4 ± 11.7	73.3 ± 12.1	66.0 ± 13.7
3	16.8 ± 5.0	17.0 ± 5.6	8.1 ± 3.3
4	3.7 ± 1.1	3.3 ± 2.2	1.7 ± 1.3
*Note*. UN-OP = unoperated; VEH = vehicle solution; SAP = saporin. The total time spent on each alley is presented as the mean for each group ± the standard error of the mean (SEM) in seconds. Data were subjected to a log_10_ transform prior to repeated measures ANOVA analysis. There was no Group × Alley interaction.
